# Sweet’s Syndrome in a Patient With Seropositive Rheumatoid Arthritis After Starting Adalimumab: Is Sweet’s Syndrome Related to Rheumatoid Arthritis or Is It the Paradoxical Effect of Adalimumab?

**DOI:** 10.7759/cureus.16804

**Published:** 2021-08-01

**Authors:** Prodip Paul, Chad P Walker, Mishouri Paul, Dipon Dey

**Affiliations:** 1 Internal Medicine, Geisinger Community Medical Center, Scranton, USA; 2 Rheumatology, Geisinger Musculoskeletal Institute, Scranton, USA; 3 Rheumatology, Geisinger Health System, Scranton, USA; 4 Medicine, Interfaith Medical Center, New York, USA; 5 Epidemiology and Public Health, ZWH Medical Care PC, New York, USA

**Keywords:** sweet's sydrome, adalimumab, rheumatoid arthritis, anti-tnf-alpha, neutrophilic dermatosis

## Abstract

Sweet’s syndrome is a rare acute febrile neutrophilic dermatosis accompanied by fever, neutrophilia, and asymmetrical distribution of tender erythematous skin lesions. The underlying biological pathways responsible for this inflammatory skin disorder are not yet clearly established. However, an association with autoimmune disease, neoplasm, and drugs could be indicative of unusual hypersensitivity involving proinflammatory cytokines. There are several case reports indicating an association between Sweet’s syndrome and rheumatoid arthritis (RA). Proinflammatory cytokines are considered to play a vital role in the pathogenesis of both RA and Sweet’s syndrome. Adalimumab works against proinflammatory cytokines and is considered a disease-modifying antirheumatic drug in RA; it is also reported to be effective in refractory Sweet’s syndrome. While adalimumab has been proven to be beneficial in autoimmune disorders and inflammatory conditions, there are also reports of paradoxical development of Sweet’s syndrome with adalimumab. In this report, we present a case of Sweet’s syndrome in a 74-year-old adult patient with a history of seropositive RA who developed Sweet’s syndrome within two months after the initiation of adalimumab therapy.

## Introduction

Sweet's syndrome was first described by Dr. Robert Douglas Sweet in 1964 [1]. It is also known as acute febrile neutrophilic dermatosis and is characterized by rapid onset of fever, asymmetrically distributed tender erythematous papules, nodules, and plaques, and usually involves the face, neck, and upper extremity. Dermal infiltration of mature neutrophils in the upper dermis is responsible for acute inflammatory skin eruption. Based on the clinical settings, Sweet’s syndrome can be classified into three subgroups: classical, malignancy-associated, and drug-induced [2].

Classical Sweet's syndrome (CSS), also known as idiopathic Sweet’s syndrome, is more common in women aged 30-50 years and is often preceded by an upper respiratory tract infection [2]. It is reported to be associated with inflammatory bowel disease with colonic involvement [3] and pregnancy [4]. Malignancy-associated Sweet's syndrome (MASS) occurs as a paraneoplastic syndrome in Sweet’s syndrome-related hematologic malignancy or undiagnosed visceral malignancy [2]. Hematologic malignancies such as acute myelogenous leukemia [5], myelodysplastic syndrome (MDS) [6], and multiple myeloma [7] have been reported with Sweet’s syndrome. Drug-induced Sweet’s syndrome (DISS) is reported to be associated with granulocyte-colony stimulating factor, tretinoin, trimethoprim-sulfamethoxazole (TMP-SMX), azathioprine, bortezomib, imatinib [2], and ipilimumab [8].

Sweet’s syndrome has also been reported with RA, Behçet’s disease, relapsing polychondritis, sarcoidosis, Grave’s disease, Hashimoto’s thyroiditis, and systemic lupus erythematosus [2]. T-cell-associated cytokine release may be responsible for the pathogenesis of Sweet’s syndrome. Cytokine release leads to neutrophil infiltration, accumulation, and activation in the affected site [9]. Systemic corticosteroids, potassium iodine, and colchicine are the first-line treatments for Sweet’s syndrome, while indomethacin, clofazimine, cyclosporine, and dapsone have been reported as effective second-line drugs [10]. Tumour necrosis factor α (TNF-α) inhibitor [11] and interleukin 1 inhibitors [12] have also been reported to be effective in the treatment of refractory Sweet’s syndrome.

While anti-TNF agents have been proven to be beneficial in autoimmune disorders and inflammatory conditions, these agents can be associated with paradoxical development of psoriasis [13] and Sweet’s syndrome [14]. In this case report, we discuss a case of a 74-year-old male with a history of seropositive RA who developed Sweet’s syndrome two months after the initiation of adalimumab therapy.

## Case presentation

**Figure 1:**
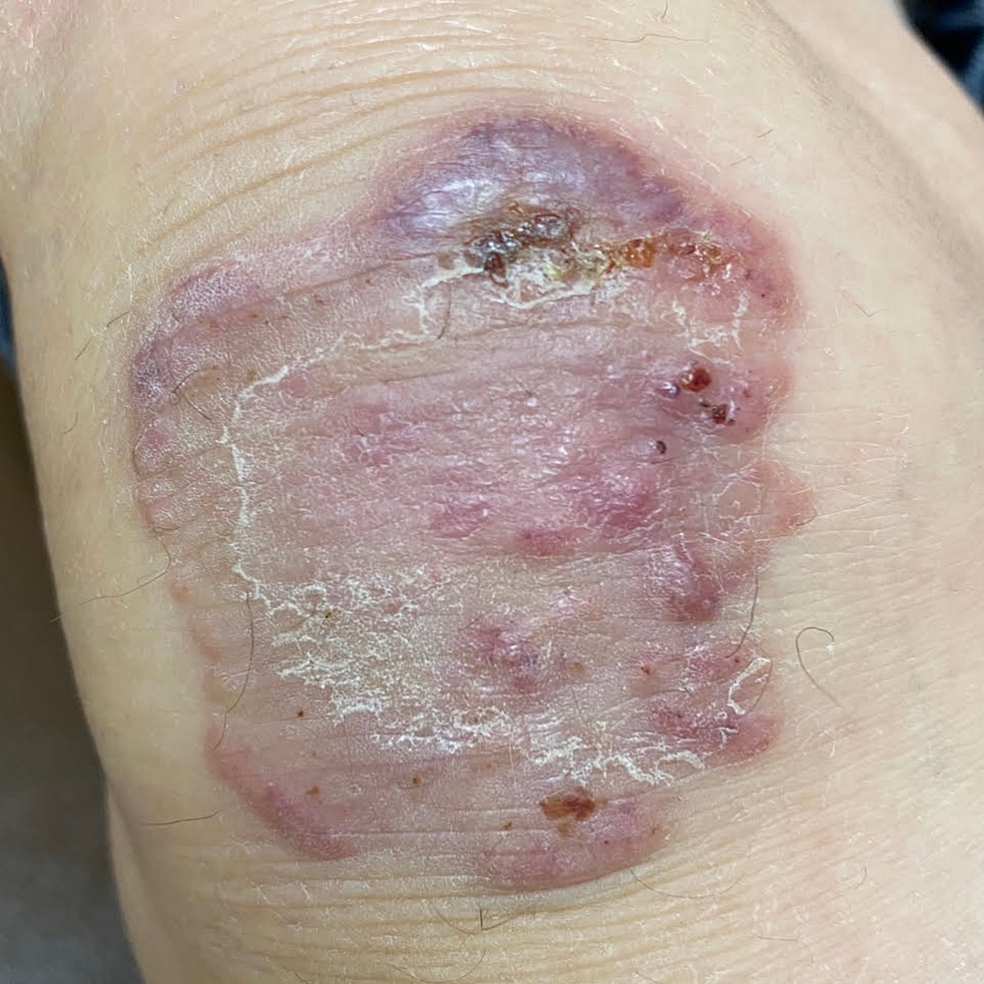
Pink/purple raised irregular texture of skin lesions of about 5 cm over the right knee

**Figure 2:**
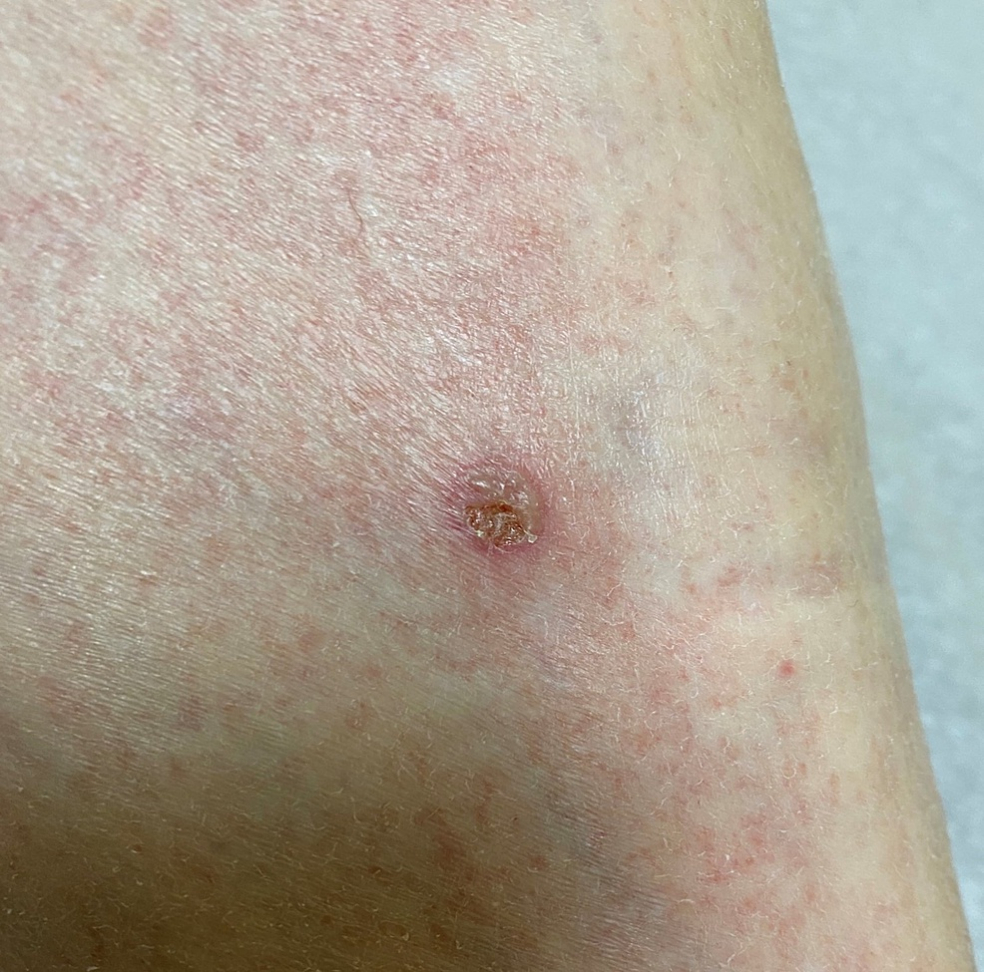
Round pink plaque over the left shin

**Figure 3:**
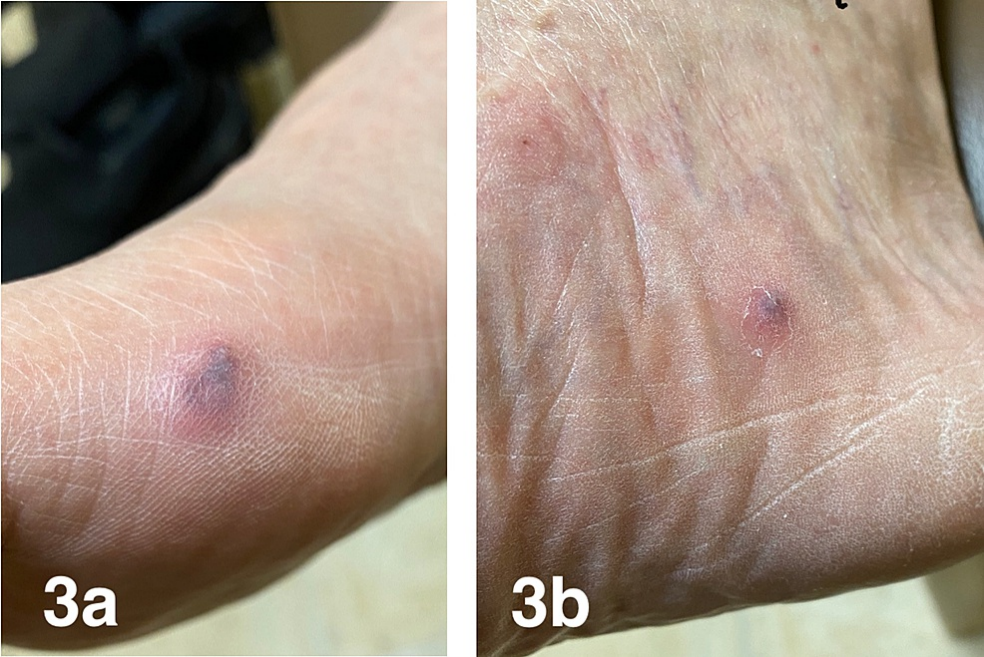
Two areas (3a and 3b) of similar dry, purple discolored skin lesions over the medial aspect of the right foot

A 74-year-old male with a past medical history of hypertension, diabetes mellitus type 2, and benign prostatic hypertrophy was diagnosed with seropositive RA (rheumatoid factor and anti-cyclic citrullinated peptide-positive) three years ago. He had an initial clinical response to methotrexate but developed active inflammatory symptoms about a year into the treatment, which responded to short courses of steroids. Therefore, hydroxychloroquine was added to his regimen. He was also started on adalimumab about two years after the initial presentation. The patient had significant improvement of inflammatory joint symptoms after the first dose of adalimumab. However, after four doses, he started to have skin eruptions on his right knee, lower legs, feet, and right elbow. On examination, he had about 5-cm, pink/purple raised irregular texture of skin lesions over the right knee (Figure 1), round pink plaque over the left shin (Figure 2), two areas of similar dry, purple discolored skin lesions over the medial aspect of right foot (Figures 3a, 3b), two raised, round skin lesions with central, superficial ulceration over the right elbow, 1.5 cm in dimension with a boggy collection of fluid over right second PIP.

**Figure 4:**
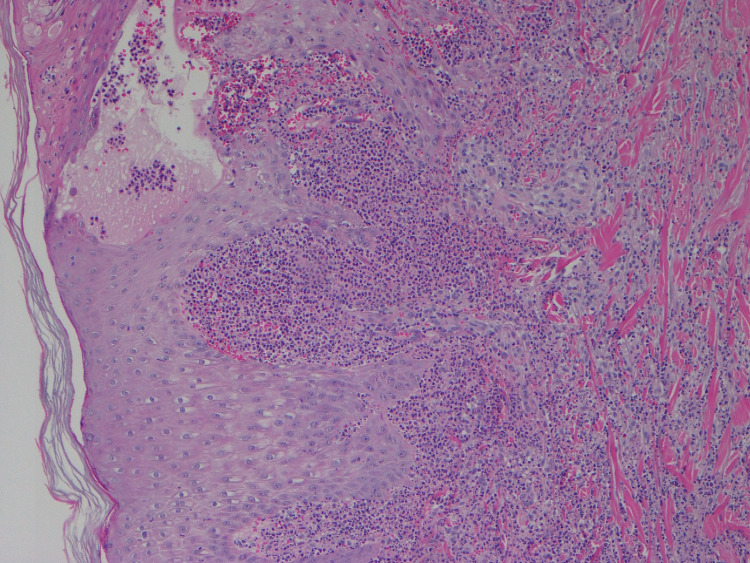
Skin biopsy (H&E stain at 100x) showing necrotizing neutrophilic dermatosis H&E: hematoxylin and eosin

The patient reported pain on his feet at the skin eruption site. He did not have recurring inflammatory joint pain. Adalimumab and hydroxychloroquine were discontinued, and he was started on an oral steroid taper with outpatient dermatology follow-up. Skin biopsy revealed necrotizing neutrophilic dermatosis (Figure 4), consistent with Sweet’s syndrome.

The patient had a relapse of skin lesions about four months after completing a steroid taper. He was then started on colchicine with good response but had persistent gastrointestinal (GI) intolerance. He was started on dapsone 25 mg daily and had a good response. The dose had to be increased to 50 mg daily due to recurring lesions. This was effective for his skin lesions.

The patient remained off adalimumab. When he developed symptoms of active RA, he was started on upadacitinib with a good clinical response.

## Discussion

Sweet’s syndrome presents as an acute febrile neutrophilic dermatosis with a constellation of clinical symptoms, physical features, and pathological findings including fever, neutrophilia, and asymmetrically distributed painful tender erythematous skin lesions, consisting of papules, nodules, and plaques [2]. In our case, the patient presented with skin eruptions on his right knee, lower legs, feet, and right elbow. He also reported pain in his feet at the skin eruption site. Inflammatory dermatosis with skin eruption and pain can be explained by increased proinflammatory cytokines, which has been reported as the probable biologic mechanism responsible for Sweet’s syndrome [2].

Sweet’s syndrome can appear before or concurrent with the development of hematologic malignancy [2]. Moreover, it has been associated with inflammatory bowel disease with colonic involvement [3]. Our patient was evaluated by his primary care physician for malignancy screening but he declined a colonoscopy.

Sweet’s syndrome has been reported as a manifestation of RA [2]. Our patient was diagnosed with seropositive RA three years prior. The patient did not have recurring inflammatory joint pain while he was on hydroxychloroquine and adalimumab. However, he developed skin eruptions after four doses of adalimumab. Though adalimumab helped him to control inflammatory joint pain, it might have caused a paradoxical effect triggering the development of Sweet’s syndrome. Case reports have been published about the development of Sweet’s syndrome after the initiation of adalimumab, a TNF-α inhibitor [14,15]. TNF-α is reported as an important cytokine mediator for Sweet’s syndrome [16]. Anti-TNF-α therapy has been proven effective in Sweet’s syndrome with coexisting inflammatory bowel disease, Sjogren’s syndrome [16], and RA [17]. Yang et al. have reported tyrosine kinase inhibitor-induced Sweet’s syndrome with a median of two months' latency period [18]. Our patient developed Sweet’s syndrome two months after the initiation of adalimumab. In this case, Sweet’s syndrome could be associated with underlying RA. However, Sweet’s syndrome as a paradoxical effect of adalimumab cannot be ruled out due to the temporal relationship between the initiation of adalimumab and the development of Sweet’s syndrome.

Systemic corticosteroid is the gold standard for the treatment of Sweet’s syndrome, resulting in prompt response consisting of dramatic improvement of both the skin lesions and dermatosis-related symptoms. In case of relapse or recurrence of Sweet’s syndrome, colchicine is an effective first-line alternative, while dapsone can be considered as second-line. Recurrence of Sweet’s syndrome is quite common with variable episodes of remission. Remission can occur either after spontaneous remission or therapy-induced clinical resolution [19]. In our case, the patient initially responded to oral prednisone. However, he had two relapses after therapy-induced clinical resolution. The patient was later started on colchicine, which he could not tolerate due to GI symptoms, and was switched to dapsone. He had an excellent response to dapsone.

## Conclusions

Sweet’s syndrome has been reported in RA, which is commonly treated with anti-TNF-α agents. Anti TNF-α agents have also been used successfully to treat Sweet’s syndrome. Sweet’s syndrome occurring soon after the initiation of adalimumab could be indicative of a paradoxical effect in predisposed individuals. Because of the widespread use of adalimumab in RA patients and its potential association with Sweet’s syndrome, physicians should consider Sweet’s syndrome in such patients. More studies on a molecular basis are required to better understand the etiology of this disease, which is crucial for selecting the optimum treatment.
